# An evidence mapping and analysis of registered COVID-19 clinical trials in China

**DOI:** 10.1186/s12916-020-01612-y

**Published:** 2020-06-01

**Authors:** Liming Lu, Fan Li, Hao Wen, Shuqi Ge, Jingchun Zeng, Wen Luo, Lai Wang, Chunzhi Tang, Nenggui Xu

**Affiliations:** 1 Clinical Research and Data Center, South China Research Center for Acupuncture and Moxibustion, Medical College of Acu-Moxi and Rehabilitation, Guangzhou University of Chinese Medicine, Guangzhou, China; 2 Department of Biostatistics, Yale School of Public Health, New Haven, CT USA; 3 Center for Methods in Implementation and Prevention Science, Yale School of Public Health, New Haven, CT USA; 4 Department of Acupuncture, First Affiliated Hospital of Guangzhou University of Chinese Medicine, Guangzhou, China; 5 School of Medical Information Engineering, Guangzhou University of Chinese Medicine, Guangzhou, China

## Abstract

**Background:**

This article aims to summarize the key characteristics of registered trials of 2019 novel coronavirus (COVID-19), in terms of their spatial and temporal distributions, types of design and interventions, and patient characteristics among others.

**Methods:**

A comprehensive search of the registered COVID-19 trials has been performed on platforms including ClinicalTrials.gov, WHO International Clinical Trials Registry Platform (WHO ICTRP), Chinese Clinical Trials Registry (CHiCTR), Australian Clinical Trials Registry, Britain’s National Research Register (BNRR), Current Control Trials (CCT), and Glaxo Smith Kline Register. Trials registered at the first 8 weeks of the COVID-19 outbreak are included, without language restrictions. For each study, the registration information, study design, and administrator information are collected and summarized.

**Results:**

A total of 220 registered trials were evaluated as of February 27, 2020. Hospital-initiated trials were the majority and account for 80% of the sample. Among the trials, pilot studies and phase 4 trials are more common and represent 35% and 19.1% of the sample, respectively. The median sample size of the registered trials is 100, with interquartile range 60–240. Further, 45.9% of the trials mentioned information on a data monitoring committee. 54.5% of the trials did not specify the disease severity among patients they intend to recruit. Four types of interventions are most common in the experimental groups across the registered studies: antiviral drugs, Traditional Chinese Medicine (TCM), biological agents, and hormone drugs. Among them, the TCM and biological agents are frequently used in pilot study and correspond to a variety of primary endpoints. In contrast, trials with antiviral drugs have more targeted primary outcomes such as “COVID-19 nucleic acid test” and “28-day mortality.”

**Conclusions:**

We provide an evidence mapping and analysis of registered COVID-19 clinical trials in China. In particular, it is critical for ongoing and future studies to refine their research hypothesis and better identify their intervention therapies and the corresponding primary outcomes. It is also imperative for multiple public health divisions and research institutions to work together for integrative clinical data capture and sharing, with a common objective of improving future studies that evaluate COVID-19 interventions.

## Background

An ongoing outbreak of 2019 novel coronavirus disease (COVID-19) poses a major challenge for public health [1–3]. The cumulative number of reported cases is still climbing as of the writing of this article [4–6]. However, at present, there is no confirmed effective treatment strategy for COVID-19.

The rapid spread of COVID-19 in China and the widespread fear make it high priority to explore potential intervention strategies to control the disease outbreak. Development for candidate therapeutics and vaccines has been prioritized in COVID-19 research set by world experts and funders [7]. The sooner the clinical trials are initiated, the higher the chance that an efficacious and safe intervention can be identified. Since the outbreak of COVID-19, the number of COVID-19 trials registered on various trial registry platforms worldwide has been rapidly increasing.

Designing clinical trials for COVID-19 during the spread of an epidemic comes with challenges, including scientific rigor in methodology, best ethical practice, and time considerations [8]. To date, information regarding basic characteristics and design methodologies of registered trials on COVID-19 is scarce, and little relevant literature of clinical trial methodology on COVID-19 has been published. To review current practices and identify knowledge gaps in designing clinical trials on COVID-19, we extract a relevant set of registered trials and summarize their key characteristics and design issues. We further visualize the relationship between test drugs, outcomes, and disease severity to assess the consistency of these design factors. Finally, we offer suggestions to address potential issues in future designs of such trials. The purpose of this article is to provide a contemporary list of references for COVID-19 trials and discuss their implications for ongoing and future trials. The findings of this article could shed light on missed opportunities, encourage future studies to consider more rigorous methodology, and ultimately accelerate the research process to help control the disease outbreak.

## Methods

### Search strategy

We perform a comprehensive search of the registered trials of COVID-19. Two investigators (WL and LW) have searched ClinicalTrials.gov, WHO International Clinical Trials Registry Platform (WHO ICTRP), Chinese Clinical Trials Registry (CHiCTR), Australian Clinical Trials Registry, Britain’s National Research Register (BNRR), Current Control Trials (CCT), and Glaxo Smith Kline Register from January 1, 2020, to February 27, 2020, without language restrictions. They have worked together to identify sets on different sources, before merging the results. The keywords used in the search include “2019-nCoV,” “COVID-19,” “pneumonia,” and “SARS-CoV-2.”

### Selection criteria

Registered trials are considered eligible if they meet all of the following inclusion criteria: (i) each study must include a potential treatment or intervention strategy of COVID-19 and (ii) each study should be either an interventional (clinical trials) or an observational study. No restrictions were set on patient age, trial design, and types of interventions in the experimental and control groups.

According to Guideline on Diagnosis and Treatment of COVID-19 (version 6) [9], patient disease severity can be divided into the following types: (1) mild—the clinical symptoms were mild, and no pneumonia found in imaging; (2) common—with fever, respiratory tract and other symptoms, and pneumonia found in imaging; (3) severe—comply with any of the following: (A) in case of shortness of breath, respiratory rate ≥ 30 times/min; (B) in the state of rest, finger oxygen saturation ≤ 93%; and (C) arterial oxygen partial pressure (PaO_2_)/inspired oxygen (FiO_2_) ≤ 300 mmHg; and (4) critical—has at least one of the following symptoms: (A) respiratory failure requiring mechanical ventilation, (B) shock, and (C) other organ failure requiring ICU monitoring and treatment.

### Outcomes

Among the COVID-19 trials, the endpoints selected are rate of lung imaging recovery, oxygenation index, COVID-19 nucleic acid test, 28-day mortality and length of hospital stay (days), lower Murray lung injury score, time to clinical improvement, the incidence of composite adverse outcome, the difference of PaO_2_/FiO_2_, rate of no fever, and rate of disease remission.

### Data extraction

We use a predesigned spreadsheet to collect study data. Eligibility of registered trials is determined by two reviewers (WL and LW), who also independently extract the data. Ambiguous trials are examined by a third reviewer (LL). Disagreements are resolved by discussion. All the following information is extracted from each study: (1) tracking information—actual start date of the study; (2) descriptive information—study type, study phase, and length of study time; (3) study design—interventional study (randomization allocation, intervention, masking, primary outcomes, and secondary outcomes), observational study (model, time perspective), number of arms, trial medications, inclusion criteria, exclusion criteria, and disease severity; (4) recruitment information—recruitment status, enrollment information, estimated completion date of the study, sex/gender and ages of the target population, and location; and (5) administrative information—trial registry number, primary study sponsor, trial initiator, and collaborators.

### Data summarization and visualization

Evidence mapping is used to describe the basic characteristics of the registered trials of COVID-19 in terms of their spatial, temporal, and other characteristic distribution. Curve chart is applied to describe the temporal distribution, of which the horizontal axis indicates the time range (the first 8 weeks of the COVID-19 outbreak) and the vertical axis indicates the number of registered trials. The geographic map is used to describe the spatial distribution (deeper color represents a large number of registered trials). Based on the characteristics of the registered trials of COVID-19, we create a multidimensional plot to visualize the relationship among test drugs, outcomes, and disease severity. Throughout, percentage frequency distribution is used to describe categorical data.

### Role of the funding source

The funder of the study has no role in the study design, data collection, data analysis, data interpretation, or writing of the report. The corresponding authors have full access to all the data in the study and maintain responsibility for the decision to submit for publication.

## Results

**Fig. 1:**
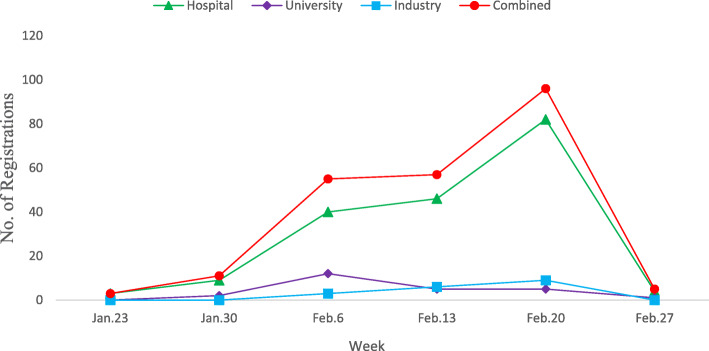
Number of new trials registered in trial registry platforms each week and by initiators. The number of new registrations per week (beginning on the date indicated) from January 23, 2020, to February 27, 2020. The “Industry” category includes all commercial data providers; the “University” category includes university and college data providers. The “combined” category combines the numbers of registrations in “Hospital,” “University,” and “Industry” categories

A total of 388 registered trials are identified on all trial registry platforms. We exclude duplicates and confirm that each of the included studies focuses on disease directly relevant to COVID-19. Two independent reviewers perform the screening, and the final set includes 220 registered trials. Details of the screening process are provided in Additional file 1: Figure S1. Figure 1 presents the number of new trials registered each week and by initiators. While only a few studies were recorded in clinical trial register platforms by January 23, 2020, the number of registered trials quickly accumulated to 220 as of February 27, 2020. Particularly, around 80 trials were initiated by hospitals during the week of February 20, 2020.

### General characteristics of registered trials

**Fig. 2:**
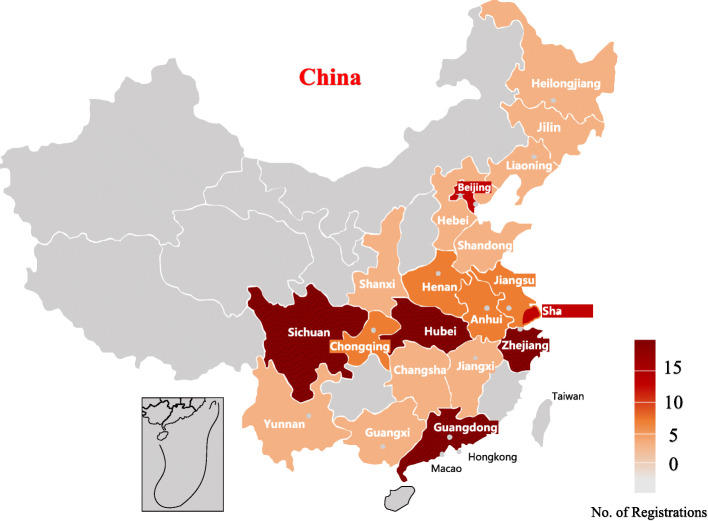
Number of registered trials distributed in each area

We summarize the general characteristics of the included trials in Table 1. Within our final set, 159 (72.3%) are intervention trials and 61 (27.7%) are observational trials. Compared to university- (19, 8.6%) and industry-initiated trials (20, 9.1%), hospital-initiated trials represent the majority (176, 80%) (also see Fig. 1). Over half of the trials (124, 55.5%) are in recruiting status, and only 1 study is completed. Regarding the length of study, 77 (35%) trials anticipate to finish within 3 months, while more than half (111, 50.5%) specify study duration between 3 and 12 months. Most studies exclusively focus on adult patients. For example, 135 (61.5%) trials intend to recruit only patients aged 18 years or older, while 30 (13.7%) studies allow patients younger than 18 years old (only 2 trials exclusively focus on patients younger than 18, which indicates a lack of pediatric trials). A total of 197 (89.5%) trials are initiated in China, while 23 (10.5%) studies do not provide such information. Among the trials initiated in China, the majority is located in Wuhan (89, 40.5%) (also see Fig. 2 for the geographical distribution of the registered studies). Seventy-one (32.3%) trials have regional collaborations only within China, and 4 (1.8%) trials have international collaborations with the USA or France, while the majority (145, 65.9%) does not provide collaboration information. Finally, about half of the trials (101, 45.9%) included a data monitoring committee.

**Table 1:** General characteristics of the included trials

	Number	Percentage
Study type		
Interventional	159	72.3
Observational	61	27.7
Study initiator		
Hospital	176	80.0
Industry	20	9.1
University	19	8.6
Other	5	2.3
Recruitment status		
Not yet recruiting	97	44.0
Recruiting	122	55.5
Completed	1	0.5
Length of study time		
L ≤ 3m	77	35.0
3 < L ≤ 6m	44	20.0
6 < L ≤ 12m	67	30.5
12 < L ≤ 24m	23	10.5
L > 24	2	0.9
NP	7	3.1
Age group of the recruited or intended population		
0–1	1	0.5
0–18	1	0.5
3 years and older	1	0.5
12 years and older	1	0.5
14 years and older	5	2.3
15 years and older	2	0.9
16 years and older	4	1.8
18 years and older	132	60.0
22 years and older	1	0.5
30 years and older	1	0.5
60 years and older	1	0.5
All	15	6.8
NP	55	25.0
Locations		
China	197	89.5
NP	23	10.5
Collaborators		
Chinese collaborators	71	32.3
International collaborators	4	1.8
NP	145	65.9
Data monitoring committee		
Has data monitoring committee	101	45.9
Not have data monitoring committee	22	10.0
Not yet determined	47	21.4
NP	50	22.7
Study start date (year, month)		
2020, January	34	15.5
2020, February	180	81.8
2020, March	6	2.7
Study sponsor		
Government	65	29.5
Government and industry	2	0.9
Government and hospital	1	0.5
Hospital	44	20.0
Hospital and industry	2	0.9
Hospital and university	1	0.5
Industry	13	5.9
University	14	6.4
NP	78	35.5
Ethical approval		
Obtained	142	64.5
Non-obtained	39	17.7
NP	39	17.7
Number of research centers		
Single center	104	47.3
Multicenter	79	35.9
NP	37	16.8

*m* month, *NP* not provided

### Methodology issues of registered trials

Table 2 summarizes the design characteristics of the registered trials. Among the interventional studies, 77 (35%) are pilot study, 2 (0.9%) in phase 1, 5 (2.3%) in phase 2, 6 (2.7%) in phase 3, and 42 (19.1%) in phase 4. There are 107 (48.6%) trials with two comparison groups, while 42 (19.1%) trials have three or more comparison groups The majority (151, 68.6%) adopt the parallel design, and 17 (7.7%) adopt the factorial design. A total of 122 trials (55.5%) use randomization, among which 68 (55.7%) indicate the use of computer software to generate the randomization algorithm. Only 23 trials (10.5%) are masked. The median sample size of the registered trials is 100, with interquartile range 60–240. In particular, 112 (50.9) trials include no more than 100 total patients. Finally, more than half of the trials (120, 54.5%) do not specify the disease severity among patients they intended to recruit.

**Table 2:** Design of registered trials

	Number	Percentage
Trial phase		
Pilot study	77	35.0
Phase 1	2	0.9
Phase 1/phase 2	3	1.4
Phase 2	5	2.3
Phase 2/phase 3	6	2.7
Phase 3	6	2.7
Phase 4	42	19.1
NP	79	35.9
Number of arms		
0	2	0.9
1	49	22.3
2	107	48.6
3	25	11.4
4	10	4.4
5	5	2.3
6	1	0.5
8	1	0.5
NP	20	9.1
Study design type		
Parallel design	151	68.6
Factorial design	17	7.7
NP	52	23.6
Randomization		
Randomized	122	55.5
Computer software	68	55.7
Phone/WeChat	1	0.8
NP	53	43.4
Non-randomized	73	33.2
NP	25	11.4
Masking (blinding)		
Blinding	23	10.5
Participant	5	21.7
Investigator	1	4.3
Investigator, outcomes assessor	1	4.3
Participant, care provider, outcomes assessor	1	4.3
Participant, care-provider, investigator, outcomes assessor	4	17.4
NP	11	47.8
Non-blinding	67	30.5
NP	130	59.0
Total sample size		
0–100	112	50.9
101–200	44	20.0
201–300	17	7.8
> 300	47	21.3
Severity of illness		
Mild	2	0.9
Mild/common	14	6.4
Mild/severe	2	0.9
Mild/common/severe	8	3.6
Mild/common/critical	1	0.5
Common	27	12.3
Common/severe	6	2.7
Common/severe/critical	3	1.4
Severe	24	10.9
Severe/critical	7	3.2
Critical	6	2.7
NP	120	54.5
The number of major outcome		
One	104	47.2
Two	37	16.8
Three	31	14.1
More than three	47	21.4
NP	1	0.5
Control intervention type		
Positive drug	14	6.4
Positive drug and conventional therapy	3	1.4
TCM	3	1.4
TCM and positive drug	1	0.5
Placebo	19	8.6
No treatment	6	2.7
Conventional therapy	89	40.5
NP	85	38.6
Intervention type of experimental group		
Antiviral drug	37	16.8
Biologicals	22	10.0
Hormone drug	7	3.2
Compound Chinese herbal medicine	24	10.9
Traditional Chinese Medicine injection	5	2.3
TCM and conventional western medicine	26	11.8
TCM and antiviral drug	1	0.5
Biologicals and conventional treatment	10	4.5
Antiviral drug and hormone drug	2	0.9
Antiviral drug and biologicals	3	1.4
Antiviral drug and conventional treatment	3	1.4
Antiviral drug and biologicals and hormone drug	1	0.5
Antiallergic and conventional treatment	1	0.5
Anti-inflammatory drug	2	0.9
Vitamin C	2	0.9
Sedative	2	0.9
Drug to regulate intestinal flora and conventional treatment	1	0.5
Daoyin + conventional treatment	1	0.5
Acupoint stimulation and qigong and conventional treatment	1	0.5
TCM and moxibustion	1	0.5
NP	68	30.9

*NP* not provided, *TCM* Traditional Chinese Medicine

### Types of interventions in registered trials

Additional file 1: Figure S2 summarizes the number of new trials registered in trial platforms each week by different types of test drugs. Overall, the Traditional Chinese Medicine (TCM) is frequently included in the registered trials, while the hormone drugs are the least tested. Additional file 1: Table S1 includes a more detailed description of primary drugs in the trials. In the experimental groups, TCM (78, 35.5%) is frequently included as the intervention. Further, lopinavir/ritonavir (9, 15%), Arbidol (7, 11.7%), chloroquine phosphate tablets (5, 8.3%), and hydroxychloroquine (4, 6.7%) are the most frequently seen in the antiviral drug category. In contrast, a diverse list of drugs are considered in the biological agents category and the hormone drugs category. In the control group, the conventional therapy, active drugs, and placebo appear most frequently.

### Relationship between test drugs, outcomes, and disease severity

**Fig. 3:**
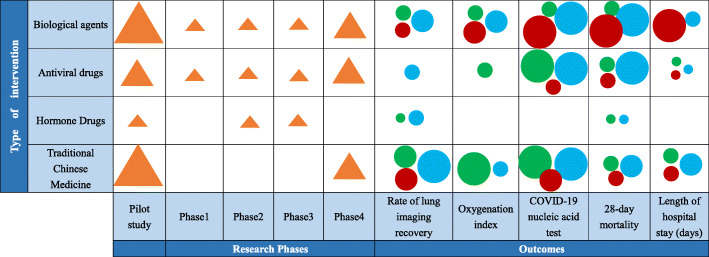
A multidimensional plot for the relationship between test drugs, outcomes, and disease severity. Each circle or triangle represents one type of intervention specified in the corresponding row. (1) Circle and triangle size: number of studies evaluating each intervention (larger = more studies). (2) Circle color: stage of disease (green for mild, blue for moderate, red for severe). Biological agents: interferon alfa, thymosin, vMIP, stem cell-based medicinal products, novaferon, plasma therapy, tocilizumab, camrelizumab, and immunoglobulin. Antiviral drugs: Arbidol tablets, chloroquine phosphate tablets, ritonavir, lopinavir, hydroxychloroquine, chloroquine, baloxavir marboxil, emtricitabine, polyinosinic-polycytidylic acid injection, favipiravir, oseltamivir, remdesivir, darunavir, cobicistat, and ribavirin. Hormone drugs: methylprednisolone. TCM: clearing lung formula (Qing Fei Fang), ginseng, poria and *Atractylodes macrocephalae* powder (Shēn Líng Bái Zhú Săn), instant relief for cough syrup (Ke Su Ting Tang Jiang), Chinese medicine formulas 1 and 2 (Zhong Yao 1 Hao and 2 Hao), cough cleared capsules (Ke Qing Capsules), clearing both common cold and cough capsules (Gan Ke Shuang Qing Capsules), antiviral oral liquid, the Radix Fici Hirtae preventing COVID-19 formula (WuZhi Fang Guan Fang) antiviral particles, YinHu QingWen decoction, relief for heat and toxin injection (Re Du Ning Zhu She Ji), relief for inflammation injection (Xi Yan Ping Injection), ginseng and Radix Astragali reinforcing health injection (Shen Qi Fu Zheng Injection), and others not provided

In our review, we have identified that some researchers define multiple indicators (3 or more) as the primary outcome when they enter the registration information. We summarize these results truthfully (without picking one among the multiple primary outcomes) and find that the most frequent primary outcomes are “Rate of lung imaging recovery,” “Oxygenation index,” “COVID-19 nucleic acid test,” “28-day mortality,” and “Length of hospital stay (days).” Figure 3 visualizes the relationship between test drugs, study phase, primary outcomes, and disease severity using a multidimensional plot. Evidently, TCM, antiviral drugs, and biologic agents are most frequently used in pilot studies and phase 4 trials. While antiviral drugs mainly correspond to two types of primary outcomes: “COVID-19 nucleic acid test” and “28-day mortality,” TCM and biologic agents could correspond to a variety of primary endpoints and may target multiple primary outcomes (data not shown). Figure 3 also indicates that the same primary outcome has been used to evaluate intervention for patients with different disease severity.

## Discussion

We provide an evidence mapping and analysis of global registered clinical trials, all of which aim to explore potential intervention strategies for COVID-19. We find that the number of registered clinical trials of COVID-19 treatment is growing on a weekly basis shortly after the end of January. A large fraction of registered trials are pilot studies, adopt the interventional design, and are funded by government and hospitals. As expected, the typical sample size included is not large (median sample size = 100). Drugs used in the experimental groups include antiviral drugs, TCM, biologicals, and hormone drugs, while control groups mainly include conventional therapy, active drugs, and placebo. Participants are primarily adults with mild, common, or severe COVID-19. Our findings may provide evidence for future COVID-19 studies, in terms of types of trial designs, types of drugs evaluated, types of patients included, and anticipated study duration.

Our evidence mapping also suggests that many registered trials may not have a clear research hypothesis. This is reflected by the diversity in selecting the intervention (drugs) and the primary outcomes. In Fig. 3, except for the relatively more targeted primary outcome indicators of antiviral drug (e.g., COVID-19 nucleic acid test and 28-day mortality), registered trials of TCM and biological agents usually include multiple primary outcomes. With limited information, it is unclear whether these trials intentionally include multiple co-primary outcomes, or they do not come with clear research hypotheses in the design stage. With the chaos during the early outbreak in China, little information is known about the coronavirus and the disease, which may contribute to the difficulty in refining the primary outcomes. On the other hand, researchers could consider selecting the primary outcome based on the severity of COVID-19. For example, in addition to the removal of viral infection, it seems reasonable for studies to consider mitigating fever, cough, and other symptoms for mild patients; consider addressing inflammation in imaging for common patients; and consider improving pulmonary function and survival for severe and critical patients [9]. Surrogate outcomes could also be considered. For severe and critical patients, it is more practical to observe the death of patients through 28 days in ordinary times. In the event of a health emergency, the race against the epidemic will motivate us to choose an earlier efficacy endpoint as a surrogate for mortality in order to speed up the clinical trial. For example, the primary outcome time to clinical recovery (TTCR) used by the clinical trial on remdesivir can be regarded as a surrogate endpoint [10, 11]. It evaluates the efficacy of drugs by improving clinical symptoms such as patient’s temperature, respiratory rate, and oxygen saturation.

We also find that a large fraction of the registered trials are pilot studies. More than 30% trials have at least 3 arms. The majority of trials do not mention sample size calculation, which is expected because pilot studies do not require a formal sample size calculation [12]. Despite potential limitations, these pilot studies could ultimately help generate more targeted hypotheses for future COVID-19 research at a larger scale. On the other hand, the majority of registered trials use individual-level randomization and parallel assignment. While individual randomization is the most common strategy, randomizing members of a ward can lead to unblinding or treatment contamination. The potential of cluster randomization has not yet been explored for COVID-19 studies. A cluster randomized design randomizes groups of patients to intervention arms, which may be attractive in some scenarios. However, cluster randomization requires more sophisticated considerations on the design and analysis [13, 14] and could require more administrative resources compared to individually randomized studies. Other more recent trial designs have also not yet been explored. For example, the stepped-wedge design has been considered in a previous Ebola treatment trial [8] and may be an alternative option when assignment to placebo control was not considered ethical during an epidemic. Under the stepped-wedge design, clusters are randomized to different sequences that dictate the order (or timing) at which each cluster will switch to the intervention condition [12, 15, 16]. The prospect to eventually receiving the intervention may facilitate recruitment. However, considering the lag of intervention time, this trial design is generally only suitable for the trials of mild or common patients of COVID-19. For the severe and critical patients, there may be a greater ethical concern because some administrative considerations also emerge caused by randomizing patients on the verge of death. During an epidemic, health care workers are overloaded with work and responsibility every day. Randomization and case information collection should be planned with feasibility considerations during an outbreak. With the help of international data management system, WeChat, and other modern tools, the randomization and case data entry can be more conveniently performed electronically, which also reduces the risk of virus transmission via papers. Furthermore, the collaboration between multiple public health divisions and clinical research organizations should also be prioritized to achieve integrated clinical data capture [17], and more initiatives should be launched in China towards breaking the barriers in sharing data for COVID-19 clinical research, as informed by lessons learnt in previous outbreaks [17–19]. However, our review of registered trials suggests that only 30% of the trials have regional collaborations within China, and few studies have international collaborations. The lack of interorganizational collaborations in these current trials calls for additional integrative efforts in planning of future larger trials to help us better manage the COVID-19 outbreak.

A keen reviewer has pointed out that our evidence mapping suggested a variety of uncoordinated, small, hospital-based pilot trials in China during the early outbreak. In fact, because there was little knowledge about COVID-19 in the first few weeks of the outbreak, it may have been challenging for the national authorities to develop a standard guideline for scientific research on this topic. The government may also have limited bandwidth to respond to numerous uncoordinated small trials as most of the resources have been directed to save patients’ lives and prevent the spread of disease outside of Wuhan, China. At the same time, the lack of coordination may also be reflective of the inexperience in coping with pandemics, which delayed a more coordinated approach where research network could have been established. These aspects represent important reflections from the COVID-19 outbreak and remain important lessons for China and other countries as well.

Although a large number of trials were registered soon after the end of January, these trials have not been completed during our review and thus may only provide limited evidence. Particularly, although we have extracted all registration information for clinical trials available at each registry platform, we find that not all relevant information has been reported. For example, some trials report limited information on sample size and patient disease characteristics, and therefore, we are unable to assess whether the study includes a formal sample size calculation and whether the study focused on general or specific types of COVID-19 patients. There are also a fair number of studies without any information on the study design, types of intervention, or data monitoring committee, all of which are essential for a successful trial. Therefore, the absence of such information may be indicative of poor quality of some registered trials, which could also be less likely to complete and provide useful information on efficacy. Future work is needed to follow up the progress of these trials and update the results once more information is available.

Another limitation of our study concerns the scope of this review. First, we have not included any vaccine trials. During the writing of this article, our search has not identified any registered vaccine trials on the trial registry platforms. It has been reported that several research projects on COVID-19 vaccines are ongoing [20], but the number of these ongoing trials is relatively small. Because of this, our study focuses on the therapeutic treatments. Finally, due to the timeliness, the conclusions of our study were based on the trials’ information registered in the first 8 weeks of COVID-19 outbreak. With an increasing number of registered trials in this field, the review needs to be updated to reflect more contemporary information.

## Conclusions

We provide an evidence mapping and analysis of registered COVID-19 clinical trials in China during the first 8 weeks of the outbreak. Although many trials are in the recruitment process, their distributions and key characteristics inform us the current status of clinical research in China towards potential therapeutic treatment options, and point to potential open opportunities for improving current efforts. In particular, it is critical for future studies to refine their research hypothesis and better identify their intervention therapies and the corresponding primary outcomes. These important elements may be informed by the research findings in the large number of pilot studies identified by our review. Innovative thinking of trial designs is also encouraged, which has been proven fruitful in previous Ebola trials. Finally, it is imperative for multiple public health divisions and research institutions to work together for integrative clinical data capture and sharing, with a common objective of improving future studies that evaluate COVID-19 interventions.

## Supplementary information

**Additional file 1 **: **Figure S1.** Screening process. **Figure S2.** Number of new trials registered in global registry platforms by different types of test drugs. **Table S1** Description of primary drugs in experimental and control group.

## Data Availability

The data that support the findings of this study are available from the corresponding author on reasonable request.
