# Supplementary material for: An evidence mapping and analysis of registered COVID-19 clinical trials in China
Source: BMC Med. 2020 Jun 1;18:167. doi: 10.1186/s12916-020-01612-y (PMC7268588; doi:10.1186/s12916-020-01612-y)
Supplement: Supplementary file 1 — Additional file 1 : Figure S1. Screening process. Figure S2. Number of new trials registered in global registry platforms by different types of test drugs. Table S1 Description of primary drugs in experimental and control group. [file 12916_2020_1612_MOESM1_ESM.docx]

**Supplementary Appendix**

**An evidence mapping and analysis of global registered clinical trials of COVID-19 intervention strategies**

Liming Lu^1#^, Fan Li^2,3#^, Hao Wen^1#^, Shuqi Ge^1^, Jingchun Zeng^4^, Wen Luo^1,5^, Lai Wang^1,5^, Chunzhi Tang^1*^, Nenggui Xu^1*^

1. Clinical Research and Data Center, South China Research Center for Acupuncture and Moxibustion, Medical College of Acu-Moxi and Rehabilitation, Guangzhou University of Chinese Medicine, Guangzhou, China.

2. Department of Biostatistics, Yale School of Public Health, New Haven, Connecticut, U.S.A

3. Center for Methods in Implementation and Prevention Science, Yale School of Public Health, New Haven, Connecticut, U.S.A

4. Department of Acupuncture, First Affiliated Hospital of Guangzhou University of Chinese Medicine, Guangzhou, China.

5. School of Medical Information Engineering, Guangzhou University of Chinese Medicine, Guangzhou, China.

#These authors contributed equally to this work

*Correspondence: Chunzhi Tang, [jordan664@gzucm.edu.cn](mailto:jordan664@gzucm.edu.cn); Nenggui Xu, [ngxu8018@163.com](mailto:ngxu8018@163.com)

**Catalog**

[1. Figure S1 Screening process 1](#_Toc8429)

[2. Figure S2 Number of new trials registered in global registry platforms by different types of test drugs 2](#_Toc24284)

[3. Table S1 Description of primary drugs in experimental and control group 3](#_Toc3675)

#

# **1. Figure S1. Screening process**


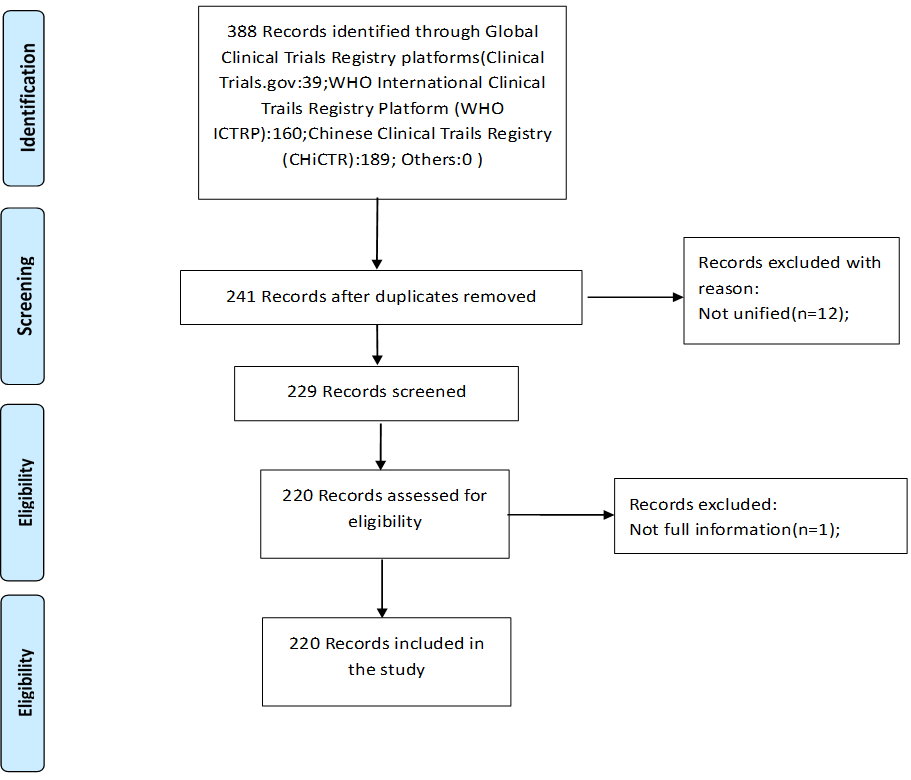


# **2. Figure S2. Number of new trials registered in trial registry platforms each week by different types of test drug**


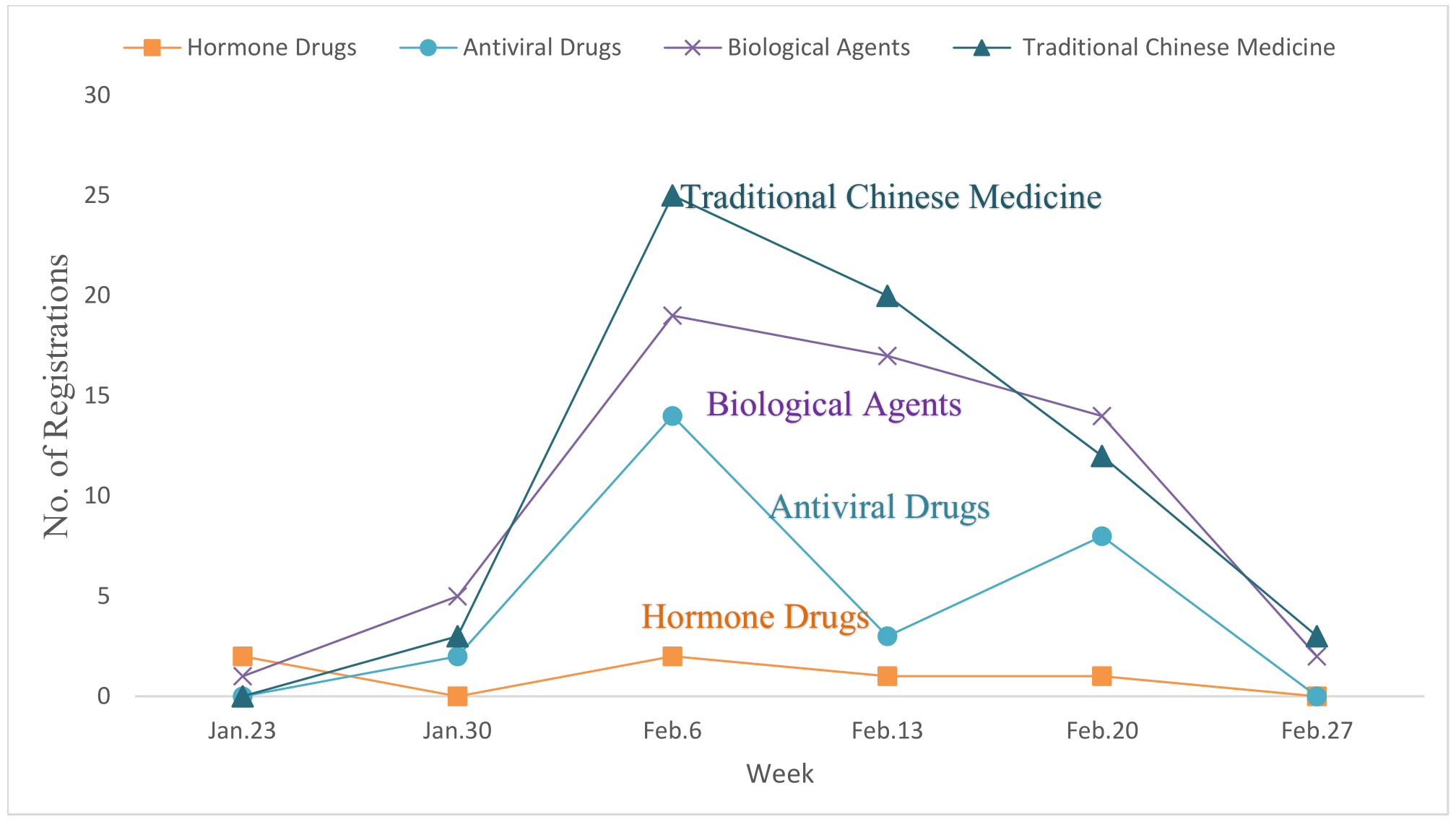


# **3. Table S1. Description of primary drugs in experimental and control group**

| **Experimental group** | | |  | **Control group** | | | |
| --- | --- | --- | --- | --- | --- | --- | --- |
| **Drugs type** | **Drugs** **name** | **Frequency**  **(Percent)** |  | **Drugs type** | **Drugs name** | | **Frequency**  **(Percent)** |
| **Antiviral drugs** |  | **60(27.3)** |  | **Antiviral drugs** |  | | **9(4.1)** |
|  | Lopinavir/[ritonavir](http://dict.youdao.com/w/ritonavir/#keyfrom=E2Ctranslation) | 9(15.0) |  |  | Lopinavir/[ritonavir](http://dict.youdao.com/w/ritonavir/#keyfrom=E2Ctranslation) | | 6(66.7) |
|  | Arbidol Tablets | 7(11.7) |  |  | Arbidol+[interferon](http://dict.youdao.com/w/interferon/#keyfrom=E2Ctranslation) | | 2(22.2) |
|  | Chloroquine Phosphate Tablets | 5(8.3) |  |  | Lopinavir/[ritonavir](http://dict.youdao.com/w/ritonavir/#keyfrom=E2Ctranslation)+[interferon](http://dict.youdao.com/w/interferon/#keyfrom=E2Ctranslation) | | 1(11.1) |
|  |  |  |  | **Biological agents** |  | | **4(1.8)** |
|  | Hydroxychloroquine | 4(6.7) |  |  | αiologicslor | | 2(50.0) |
|  | [Favipiravir tablets](http://yyws.alljournals.cn/search.aspx?subject=medicine_health&major=yx&orderby=referenced&field=key_word&q=Favipiravir+tablets) | 3（5.0） |  |  | Thymosin hymosin i | | 1(25.0) |
|  | Chloroquine | 3（5.0） |  |  | Natural killer cells and Mesenchymal Stem Cells | | 1(25.0) |
|  |  |  |  |  |  | |  |
|  |  |  |  | [**Hormone**](http://dict.youdao.com/w/hormone/#keyfrom=E2Ctranslation) |  | | **1(0.5)** |
|  | ASC09/Ritonavir | 2（3.3） |  |  | Methylprednisolone | | 1(100.0) |
|  |  |  |  | **Traditional Chinese Medicine** |  | | **2(0.9)** |
|  | Baloxavir Marboxil Tablets | 2（3.3） |  |  | YinHu QingWen Decoction | | 1(50.0) |
|  | Hydroxychloroquine Sulphate | 2（3.3） |  |  | Chinese Medicine+ Interferon | | 1(50.0) |
|  |  |  |  |  |  | |  |
|  | Remdesivir | 2（3.3） |  | **Placebo** | **/** | | **14(6.4)** |
|  | Darunavir+Cobicistat | 2（3.3） |  | **Blank** | **/** | | **9(4.1)** |
|  | GD31 | 1（1.7） |  | **Conventional** | | **/** | **90(40.9)** |
|  | Azvudine Tablets | 1（1.7） |  | **Other** | **/** | | **93(42.3)** |
|  | Danoprevir Sodium Tablets/[ritonavir](http://dict.youdao.com/w/ritonavir/#keyfrom=E2Ctranslation) | 1（1.7） |  |  |  | |  |
|  | Polyinosinic-polycytidylic Acid Injection | 1（1.7） |  |  |  | |  |
|  | Suramin Sodium | 1（1.7） |  |  |  | |  |
|  | Triazavelin(riazavelin(u | 1（1.7） |  |  |  | |  |
|  | Lopinavir/[ritonavir](http://dict.youdao.com/w/ritonavir/#keyfrom=E2Ctranslation)+[emtricitabine](http://dict.youdao.com/w/emtricitabine/#keyfrom=E2Ctranslation)+Tenofovir Alafenamide Fumarate Tablets | 1（1.7） |  |  |  | |  |
|  | ASC09F+oseltamivir | 1（1.7） |  |  |  | |  |
|  | Lopinavir/[ritonavir](http://dict.youdao.com/w/ritonavir/#keyfrom=E2Ctranslation) Tablets + "http://dict | 3（5.0） |  |  |  | |  |
|  | Prezista/cobicistat Tablets  +Thymosin hymosin | 1（1.7） |  |  |  | |  |
|  | Ribavirin+Interferon ibav | 1（1.7） |  |  |  | |  |
|  | ASC09F+Interferon | 1（1.7） |  |  |  | |  |
|  | Arbidol+Novaferon | 1（1.7） |  |  |  | |  |
|  | Lopinavir/[ritonavir](http://dict.youdao.com/w/ritonavir/#keyfrom=E2Ctranslation)+Thymosin Thymosin | 1（1.7） |  |  |  | |  |
|  | Lopinavir/[ritonavir](http://dict.youdao.com/w/ritonavir/#keyfrom=E2Ctranslation)+Novaferon | 1（1.7） |  |  |  | |  |
|  | Beta-1B  Lopinavir/[ritonavir](http://dict.youdao.com/w/ritonavir/#keyfrom=E2Ctranslation)+Interferon Beta-1B | 1（1.7） |  |  |  | |  |
|  | Arbidol+Recombinant Human Interferon  αHu | 1（1.7） |  |  |  | |  |
|  |  |  |  |  |  | |  |
|  |  |  |  |  |  | |  |
|  |  |  |  |  |  | |  |
|  |  |  |  |  |  | |  |
| **Biologics agents** |  | **43(19.5)** |  |  |  | |  |
|  | Immune Serum | 6(13.9) |  |  |  | |  |
|  | Intravenous Immunoglobulins | 3(6.9) |  |  |  | |  |
|  | Mesenchymal Stem Cells | 2(4.7) |  |  |  | |  |
|  | Umbilical Cord(UC)-Derived Mesenchymal Stem Cells(MSCs) | 2(4.7) |  |  |  | |  |
|  | Recombinant Human Interferon  αec | 2(4.7) |  |  |  | |  |
|  | Novaferon | 2(4.7) |  |  |  | |  |
|  | Granulocyte colony-stimulating factor (G-CSF) | 1(2.3) |  |  |  | |  |
|  | Intravenous Umbilical Blood Mononuclear Cell | 1(2.3) |  |  |  | |  |
|  | Camrelizumab | 1(2.3) |  |  |  | |  |
|  | Anti-aging and Active Lyophilized Powder [Particle](http://dict.youdao.com/w/particle/#keyfrom=E2Ctranslation) | 1(2.3) |  |  |  | |  |
|  | Pegasys | 1(2.3) |  |  |  | |  |
|  | Umbilical Blood Mononuclear Cell | 1(2.3) |  |  |  | |  |
|  | Umbilical [Plasma](http://dict.youdao.com/w/plasma/#keyfrom=E2Ctranslation) | 1(2.3) |  |  |  | |  |
|  | Human [Placenta](http://dict.youdao.com/w/placenta/#keyfrom=E2Ctranslation) Biologics | 1(2.3) |  |  |  | |  |
|  | Zadaxin | 1(2.3) |  |  |  | |  |
|  | Actemra | 1(2.3) |  |  |  | |  |
|  | New Genetic rSIFN-co(genetic recombinant super compound interferon) | 1(2.3) |  |  |  | |  |
|  | Meplazumab | 1(2.3) |  |  |  | |  |
|  | UC-MSCs | 1(2.3) |  |  |  | |  |
|  | PD-1 McAbs | 1(2.3) |  |  |  | |  |
|  | vMIP | 1(2.3) |  |  |  | |  |
|  | Bevacizumab | 1(2.3) |  |  |  | |  |
|  | High dose of Natural killer cells and Mesenchymal Stem Cells | 1(2.3) |  |  |  | |  |
|  | Uterine Blood Stem Cells Biologics | 1(2.3) |  |  |  | |  |
|  | Bone Marrow Mesenchymal Stem Cells(BMSCs) | 1(2.3) |  |  |  | |  |
|  | Thymosin | 1(2.3) |  |  |  | |  |
|  | Thalidomide | 2(4.7) |  |  |  | |  |
|  | Aescin | 1(2.3) |  |  |  | |  |
|  | Diammonium Glycyrrhizinate Enteric-coated Capsules | 1(2.3) |  |  |  | |  |
|  | Tranilast | 1(2.3) |  |  |  | |  |
|  | Specific Unprovided | 1(2.3) |  |  |  | |  |
|  |  |  |  |  |  | |  |
| **Hormone drugs** |  | **21(9.5)** |  |  |  | |  |
|  | [Methylprednisolone](http://dict.youdao.com/w/methylprednisolone/#keyfrom=E2Ctranslation) | 11(52.4) |  |  |  | |  |
|  | Specific Unprovided | 10(47.6) |  |  |  | |  |
| **Traditional Chinese Medicine** |  | **78(35.5)** |  |  |  | |  |
|  | Dual-yellow Oral Liquid（Shuanghuanglian oral liquid） | 2(2.6) |  |  |  | |  |
|  | Delavay Honeysuckle Decoction（*Jin Yin Hua Tang*） | 2(2.6) |  |  |  | |  |
|  | [Lianhuaqingwen capsule](http://dict.cnki.net/dict_result.aspx?searchword=%e8%bf%9e%e8%8a%b1%e6%b8%85%e7%98%9f%e8%83%b6%e5%9b%8a&tjType=sentence&style=&t=lianhuaqingwen+capsule)s | 2(2.6) |  |  |  | |  |
|  | COVID-19 Formula 1(Xin Guan Yi Hao) | 1(1.3) |  |  |  | |  |
|  | COVID-19 Formula 2(Xin Guan Er Hao) | 1(1.3) |  |  |  | |  |
|  | Yinchai Compounds granules+Qingqiao Antiviral Particles | 1(1.3) |  |  |  | |  |
|  | Consolidating the Exterior and Detoxification Particles(Gu Biao Jie Du Ling) | 1(1.3) |  |  |  | |  |
|  | Jinhao Resolving Heat Particles+Agastache Qi-Correcting Oral Liqued(Huo Xiang Zheng Qi Oral Liqued) | 1(1.3) |  |  |  | |  |
|  | Jinye Toxin-Resolving Particles Group | 1(1.3) |  |  |  | |  |
|  | Eight-Gem Composed Elixir(Ba Bao Dan) | 2(2.6) |  |  |  | |  |
|  | Dispel Pathogen and Reinforce Health Formula(Jie Duan Niu Zhuan Fang) | 1(1.3) |  |  |  | |  |
|  | Clearing the Lung and Evacuating Toxins Decoction(Qing Fei Pai Du Tang) | 1(1.3) |  |  |  | |  |
|  | Scattering Wind and Resolving Toxins Capsules(Shu Feng Jie Du Capsules) | 1(1.3) |  |  |  | |  |
|  | Clearing the Phlegm and Heat Capsules(Tan Re Qing Capsules) | 1(1.3) |  |  |  | |  |
|  |  |  |  |  |  | |  |
|  |  |  |  |  |  | |  |
|  |  |  |  |  |  | |  |
|  |  |  |  |  |  | |  |
|  | Chinese Medicine Clearing Lung Formula(Qing Fei Fang) | 1(1.3) |  |  |  | |  |
|  | Ginseng, Poria and Atractylodes Macrocephalae Powder(*Shēhowder(hai ZhúhSăn*) | 1(1.3) |  |  |  | |  |
|  | Instant Relief for Cough Syrup(Ke Su Ting Tang Jiang) | 1(1.3) |  |  |  | |  |
|  | Chinese Medicine Formula 1 and 2(Zhong Yao 1 Hao and 2 Hao) | 1(1.3) |  |  |  | |  |
|  | Cough Cleared Capsules(Ke Qing Capsules) | 1(1.3) |  |  |  | |  |
|  | Clearing Both Common Cold and Cough Capsules(Gan Ke Shuang Qing Capsules) | 1(1.3) |  |  |  | |  |
|  | Antiviral Oral Liquid | 1(1.3) |  |  |  | |  |
|  | The Radix Fici Hirtae Preventing COVID-19 Formula(WuZhi Fang Guan Fang) | 1(1.3) |  |  |  | |  |
|  | Antiviral Particles | 1(1.3) |  |  |  | |  |
|  | YinHu QingWen Decoction | 1(1.3) |  |  |  | |  |
|  | Relief for Heat and Toxin Injection(Re Du Ning Zhu She Ji) | 1(1.3) |  |  |  | |  |
|  |  |  |  |  |  | |  |
|  |  |  |  |  |  | |  |
|  | Relief for Heat and Toxin Injection(Tan Re Qing Zhu She Ji) | 1(1.3) |  |  |  | |  |
|  | Thorough Clearing for Blood Injection(Xue Bi Jing Injection) | 1(1.3) |  |  |  | |  |
|  | Relief for Inflammation Injection(Xi Yan Ping Injection) | 1(1.3) |  |  |  | |  |
|  | Radix et Rhizoma Ginseng and Radix Astragali Reinforcing Health Injection(Shen Qi Fu Zheng Injection) | 1(1.3) |  |  |  | |  |
|  | Specific Unprovided | 45(57.7) |  |  |  | |  |
|  |  |  |  |  |  | |  |
| **Others** | **Specific Unprovided** | **18(8.2)** |  |  |  | |  |
